# Microbiome associated with peri-implantitis versus periodontal health in individuals with a history of periodontal disease

**DOI:** 10.1080/20002297.2017.1325218

**Published:** 2017-06-01

**Authors:** Danae Apatzidou, David F. Lappin, Graham Hamilton, Christos A. Papadopoulos, Antonis Konstantinidis, Marcello P. Riggio

**Affiliations:** ^a^ Aristotle University of Thessaloniki, Thessaloniki, Greece; ^b^ Glasgow University, Glasgow, UK

## Abstract

The microbiome of peri-implantitis sites and healthy dental sites was determined in subjects with a history of periodontitis. Ten systemically healthy non-smokers received periodontal treatment before implant placement. Plaque samples were collected from four sites of one implant with peri-implantitis (bone loss ≥2mm; PPD ≥6mm; BOP/suppuration; >1year loading) and from one dental site per quadrant with periodontal health (PPD ≤3mm, CAL <4mm, absence of BOP and bone loss). Following DNA extraction, the bacteria present in each sample were determined by high-throughput sequencing of the V3-V4 region of the 16S-rRNA gene using the Illumina-MiSeq platform. OTUs were picked using QIIME. Differences between dental and implant sites were determined using linear discriminant analysis, effect size and diversity analyses were conducted using PAST v3.02. The microbiomes of diseased samples were less diverse than those found in health, although disease was associated with a higher abundance of taxa relative to health. The genera Actinobacillus and Streptococcus were most closely associated with health, whereas Prevotella and Porphyromonas were most discriminative for disease. Other disease associated genera were Peptostreptococcus, Tannerella, Treponema, TG5 and Atopobium. Diseased peri-implant and healthy periodontal tissues in the same individual appear to harbour distinct microbiological ecosystems with putative periodontal pathogens predominating in the peri-implantitis-associated microbiome.

